# An Interpretable Model With Probabilistic Integrated Scoring for Mental Health Treatment Prediction: Design Study

**DOI:** 10.2196/64617

**Published:** 2025-03-26

**Authors:** Anthony Kelly, Esben Kjems Jensen, Eoin Martino Grua, Kim Mathiasen, Pepijn Van de Ven

**Affiliations:** 1 Department of Electronic and Computer Engineering University of Limerick Limerick Ireland; 2 Health Research Institute University of Limerick Limerick Ireland; 3 Center for Digital Psykiatri Forskningsenhed Denmark; 4 Department of Psychology and Behavioural Sciences Aarhus University Aarhus Denmark; 5 Department of Clinical Research University of Southern Denmark Odense Denmark

**Keywords:** machine learning, mental health, Monte Carlo dropout, explainability, explainable AI, XAI, artificial intelligence, AI

## Abstract

**Background:**

Machine learning (ML) systems in health care have the potential to enhance decision-making but often fail to address critical issues such as prediction explainability, confidence, and robustness in a context-based and easily interpretable manner.

**Objective:**

This study aimed to design and evaluate an ML model for a future decision support system for clinical psychopathological treatment assessments. The novel ML model is inherently interpretable and transparent. It aims to enhance clinical explainability and trust through a transparent, hierarchical model structure that progresses from questions to scores to classification predictions. The model confidence and robustness were addressed by applying Monte Carlo dropout, a probabilistic method that reveals model uncertainty and confidence.

**Methods:**

A model for clinical psychopathological treatment assessments was developed, incorporating a novel ML model structure. The model aimed at enhancing the graphical interpretation of the model outputs and addressing issues of prediction explainability, confidence, and robustness. The proposed ML model was trained and validated using patient questionnaire answers and demographics from a web-based treatment service in Denmark (N=1088).

**Results:**

The balanced accuracy score on the test set was 0.79. The precision was ≥0.71 for all 4 prediction classes (depression, panic, social phobia, and specific phobia). The area under the curve for the 4 classes was 0.93, 0.92, 0.91, and 0.98, respectively.

**Conclusions:**

We have demonstrated a mental health treatment ML model that supported a graphical interpretation of prediction class probability distributions. Their spread and overlap can inform clinicians of competing treatment possibilities for patients and uncertainty in treatment predictions. With the ML model achieving 79% balanced accuracy, we expect that the model will be clinically useful in both screening new patients and informing clinical interviews.

## Introduction

### Overview

As in most areas of human activity, mental health care has seen an increase in the application of artificial intelligence (AI), particularly in assisting the diagnosis and treatment of mental disorders [1]. While the debate between clinical judgment and statistical methods is longstanding in the field of clinical psychology [2], under appropriate conditions, statistical tools can perform as well as—or better than—clinical judgment in diagnosing psychopathologies [3]. To be effective, these statistical tools should be context-based and simple for clinicians to use [3,4].

When assessing patients for treatment in a mental health setting, clinicians consider various screening instruments in addition to demographic and medical information, assessing the scores related to possible diagnoses and treatments based on the patient’s answers to questions [5]. Patients with complex clinical scenarios present a challenge for clinicians in assessing the various possible symptoms to arrive at a most likely diagnosis [6]. In diagnosing depression, for example, comorbidity with anxiety disorders and other factors was found to lead to uncertainty in diagnosis in as many as 40% of cases [7]. The consequences of an uncertain diagnosis may be high rates of treatment-resistant depression [7,8].

Early correct intervention may reduce the burden of disease for the individual as well as the society. The global costs of mental disorders were estimated to be US $2493 billion in 2010 and are expected to more than double by 2030 [9]. Wittchen et al [10] deemed mental disorders “the core health challenge of the 21st century.” In their key recommendations for future research, they state that “clinically sensitive and economically feasible decision algorithms could be explored to determine what types of interventions should be assigned to which type of patient.” Therefore, insight into the underlying factors that characterize a psychopathological diagnosis and the factors that discriminate between alternative diagnoses is valuable and a contribution to solving a larger and urgent health need.

Mental health care has benefited from the ability of AI technologies to analyze data, infer classifications from new data, and communicate insights from data [11]. AI has been recognized as an important tool in clinical decision-making for mental health [12]. In assessment, AI technologies offer the potential to complement the role of clinicians with diagnostic decision support technologies [13,14]. Such diagnostic tools require the AI models to be explainable and interpretable [15], both in terms of the model itself (global) and the individual model predictions (local). Interpretability refers to how a model arrives at a decision, and explainability refers to why the decision is reached [16]. While explainability in AI has been a topic of interest since the 1980s, the recent accelerations in the widespread use of AI have resulted in guidelines on the responsible use of AI. Responsible AI requires a robust model that conveys the confidence and degree of uncertainty in prediction [16-18]. Although confidence and uncertainty estimation are considered a necessity for medical AI models to enable safe clinical deployment [19], it has been claimed that most machine learning (ML) methods in the recent medical literature neglect the important issue of model uncertainty [19].

This paper presents the design of an ML model for psychopathological mental health treatment tailored to the omnipresent questionnaire-type screening instruments. The AI model described here is based on a novel hierarchical structure that aims to meet the needs of clinical interpretability while accounting for decision confidence and uncertainty. The presented approach facilitates the questionnaire instruments that are typically used in mental health care to assess symptom severity, but in a data-driven way [13], while leveraging any other information known about respondents.

This paper first lays out the rationale for explainable AI (XAI) methods in medical models, introducing the important issues of model confidence and uncertainty. Typical ways in which model confidence and uncertainty can be quantified are introduced with an emphasis on Monte Carlo dropout (MCD), the method used in this paper. The model structure is then introduced and shown to provide interpretability by design with confidence and uncertainty estimation provided through the use of MCD. Next, model training and validation on clinical data from a Danish web-based mental health treatment service are detailed and results presented. Finally, the implications of the work are discussed.

### Background

After initial interest in the explainability of AI models in the 1980s and 1990s, recent advances in AI and ML, and the increased use of such technologies in safety-critical, socioeconomically and medically impactful applications have driven a renewed interest in XAI [20]. The explainability of AI models is generally understood as the ability for a wider range of users to understand the outputs of a given AI or ML model [21]. The concept of interpretability is closely related and refers to the ability of a person to understand how a given set of inputs results in a model output [22].

As an extension to the explainability of ML models, recent endeavors have put more emphasis on responsible or trustworthy AI [23], with confidence and robustness being important technical prerequisites. The confidence of an ML algorithm is defined as the model’s own assessment that the provided inferences are correct. Robustness is defined as the ability of a model to yield consistent results in the presence of uncertainty [16]. In practice, robustness can be evaluated by perturbing the model structure to induce distribution shifts [24], introducing epistemic uncertainty. According to XAI frameworks, robustness extends explainability while increasing stability and user satisfaction [16]. Such measures of confidence and robustness are important for a model aimed at medical decision support systems (DSSs), such as the one presented in this paper [25].

### ML Methods in Relation to XAI

Logistic regression is a classification model used in ML that applies a linear combination of the data X, with weights W, to discriminate between classes of the dependent variable Y. It models the probability of an outcome using a logistic (sigmoid) function:



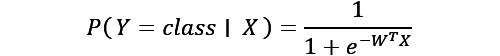



**(1)**

Since the equation consists of a linear combination of the features transformed by a sigmoid function, each weight of the model directly corresponds to the weight of a feature in determining the output. Because of this linear nature, XAI deems logistic regression to be a white-box model [16,18]. White-box models are also referred to as having the property of transparency [18]. In contrast, gray-box models and black-box models are categories of AI models with less ability to inspect the inner workings of the model (less interpretability and less transparency), but typically with more power to discriminate, and are often used in complex applications such as computer vision. This property of powerful models being black-box is often referred to as the interpretability-performance trade off [26]. Other common white-box ML models include decision trees and K-nearest neighbor models [18]. Deep neural networks are an example of a black-box model due to their many stacked nonlinear layers, typically consisting of millions of weights, which make their operation uninterpretable. Post hoc techniques add interpretability as an additional method or algorithm to the AI model [16]. One such approach is to add an additional, surrogate model that simplifies relevant sections of the AI model to be explained. Local interpretable model-agnostic explanations (LIME) [27] is a method that can explain the prediction of any classifier by building a local model that aims to approximate the original model and its predictions. The new model is chosen to be an interpretable model (such as logistic regression). This allows the user to better understand the relationship between a single input and the corresponding output prediction by providing a local explanation and is, therefore, an explanation by simplification. However, LIME has some drawbacks: it does not inherently provide global explanations of the dataset as a whole and it can experience inconsistency because it requires multiple perturbations of input data and retraining local surrogate models [28]. An alternative XAI method that ranks the importance of features used by the model is Shapley additive explanations (SHAP) [29]. SHAP is a method that calculates an additive feature importance score, which can provide local explanations for individual predictions as well as global explanations for the whole dataset. However, SHAP may experience sensitivity to feature perturbations and to feature correlations and interactions [30]. As a whole, a drawback of post hoc explainability techniques is that they approximate the model’s decision-making rather than directly reflecting its internal structure.

Since explaining black-box models requires post hoc techniques, SHAP and LIME have been applied to deep learning models in medical applications, for example, to aid the explainability of complex convolutional neural network medical diagnostic models for chest X-ray classification for COVID-19 infection [31]. SHAP has also been applied as a backend explanation module for a neural network predictor of COVID-19 infection diagnosis in eHealth record data [32], providing both local and global explanations. Post hoc XAI techniques, such as these, represent the vast majority of XAI use cases in health care [33]. To overcome the drawbacks of post hoc methods, our new approach offers an inherently interpretable model for predicting mental health treatment outcomes. Its transparency stems from a logistic regression framework, which, as described earlier, is interpretable as it is possible to make a direct link between the value of an input parameter, its associated weight, and the prediction made by the model. Moreover, by using a hierarchical structure that moves from questions to scores to classification predictions, this design enables users to understand how and why the model makes decisions, both at the questionnaire score level and the individual question level.

### Responsible AI

As previously stated, responsible AI requires an understanding of its own confidence and robustness in inferences. The advent of deep learning has resulted in a step change in the performance of models and the associated practical use of AI. However, this has come with disadvantages. Deep learning models can be confidently wrong, especially in case of a shift in the input data, and in recent years, such overconfidence in incorrect inferences has proved fatal, as illustrated by serious accidents caused by machine vision models in cars [34]. Most ML algorithms provide point-estimate predictions, meaning they provide an inference with, at best, a scalar estimate of the model’s confidence in the inference. More recently, methods based on the application of Bayesian statistics have been proposed [35]. In such methods, rather than estimating a point estimate, the models provide a probabilistic inference modeled by a probability distribution. This distribution can then be used for a point-estimate inference (eg, by taking the mode of the distribution as the inference) and can also be used to assess the model’s uncertainty in this inference by considering the shape of the probability distribution. Distributions strongly peaked around the inference indicate low uncertainty; very wide and flat distributions around the inference indicate high uncertainty.

These models often also provide probabilistic information on the effect of model predictions in the face of perturbations in the input features. For example, Cardelli et al [36] showed that for Bayesian inference with Gaussian processes, a class of algorithms overlapping with deep neural networks that have inherent uncertainty estimates [19], the model’s uncertainty in response to perturbations in the input space can be upper-bounded, thus providing a measure for the model’s robustness.

If a full Bayesian probability distribution is established for all parameters in the model, with statistics either learned from data or based on existing knowledge, it can then be used to investigate the confidence and robustness of the model. However, a significant drawback of this approach is that it can be compute-intensive [37].

An alternative method called MCD is inspired by using the dropout layers in deep neural networks to improve the ability of a network to “generalize,” or provide good quality predictions on yet unseen data [38]. When used to allow the model to generalize well, dropout sets weights of the model to 0 randomly during training only [38]. The result is that the trained model does not become overreliant on any one (potentially spurious) characteristic of the data. In contrast, MCD also uses dropout when new predictions are made. It does so by obtaining repeated inferences from the trained model, but with different weights dropped out in every iteration of the inference. The result is a distribution of inferences rather than a point-estimate. Such inference distributions can be shown to be equivalent to those obtained by a fully Bayesian model for an important family of probabilistic models, called Gaussian process models, when dropout is applied before every weight layer [39]. Consequently, MCD results in a Bayesian approximation by randomly switching off connections in the network during inference.

MCD has been applied to estimate uncertainty in medical imaging AI convolutional neural network models where the model perturbations allow the estimation of epistemic uncertainty in the prediction of potentially cancerous skin lesions [17] and brain tumors [40]. Using this method, the model is sampled N times with dropout elements set to 0 randomly according to Bernoulli random variables. Given a prediction s^*^, the model is sampled N times, with model parameters 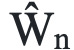 at each iteration, to yield the sample distribution y^*^. The mean value of class c (µ_c_) of the sample is the prediction probability of the class, while the variance of class c (σ_c_^2^) is the uncertainty measure:



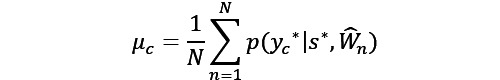



**(2)**



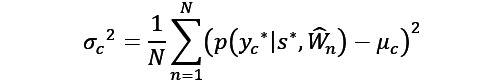



**(3)**

## Methods

### Overview

This paper presents a methodology to obtain explainable, interpretable models that are robust and provide a reliable confidence score for use with an important class of data in mental health, namely that of sum-score questionnaires. The model uses the answers to individual questions as inputs, but also explicitly represents the traditionally used sum-scores of such questionnaires in the model. Hence, explainability and interpretability are provided both at the question level and at the sum-score level. To also provide robustness and a measure of model confidence in its predictions, MCD is applied as described in the previous section.

The ability of the proposed model to provide interpretable predictions as well as estimates of confidence is demonstrated with a use case aimed at providing clinicians with treatment recommendations for several mental disorders.

### Metrics

Precision, recall, *F*_1_-score, balanced accuracy score, weighted *F*_1_-score, and area under the curve (AUC) are relevant metrics for evaluating the predictive model’s classification performance. Precision measures the proportion of correctly predicted treatment decisions among all predictions for a given class. A high precision score ensures that the model minimizes incorrect treatment assignments. Recall (or sensitivity) assesses the proportion of correctly predicted treatments among all actual instances of that class, reflecting the model’s ability to identify the correct treatment when needed. *F*_1_-score is the harmonic mean of precision and recall, balancing both metrics to provide a single performance measure, particularly useful in cases of class imbalance. The balanced accuracy score is the average of recall across all classes, accounting for imbalanced class distributions by ensuring each class contributes equally to the overall performance evaluation. Weighted *F*_1_-score adjusts the *F*_1_-score by considering class frequencies, preventing minority classes from being overshadowed in imbalanced datasets. The AUC measures the model’s ability to distinguish between different treatment classes, with higher values indicating better discrimination. During validation these metrics ensure reliable treatment predictions, minimize incorrect classifications, and balance performance across all treatment options, reducing potential clinical risks.

### Data

A dataset comprising patient questionnaire answers and demographics was used to train and validate the model. The data were gathered between November 14, 2019, and December 31, 2022, from patients of the “Internetpsykiatrien” web-based treatment services delivered by the Centre for Digital Psychiatry, Denmark. The center delivers routine care internet-delivered cognitive behavioral therapy with nationwide coverage [41]. The data consisted of answers to the Patient Health Questionnaire-9 (PHQ-9) [42], the Generalized Anxiety Disorder Questionnaire-7 (GAD-7) [43], the Social Interaction Anxiety Scale (SIAS) [44], the Panic Disorder Severity Scale (PDSS) [45], and the Fear Questionnaire [46]. Additional features included demographic information and a brief medical history, such as previously diagnosed conditions.

The ground truth of the dataset was the choice of treatment selected by the psychologists in the clinic for each patient. The treatment decision was reached using multiple steps. First, clinical psychologists or psychologists under the supervision of a clinical psychologist, assessed patients using questionnaires to determine the optimal treatment. This assessment used the same questionnaire and demographic data that was used by the model, except for free text data, which were available to the psychologists, but not to the model. Second, they performed a clinical assessment interview, including the use of the semistructured interview, Mini Neuropsychiatric Interview. Any ambiguities were discussed at a clinical conference.

A total of 91 input features were selected for inclusion in the models (Table 1), with data completeness as the primary inclusion criterion. The dataset (N=1068) was randomly split into a training set (n=801, 75%) and a test set (n=267, 25%). The training data consisted of 4 prediction class categories as follows: (1) depression (n=269, 33.6%); (2) panic (n=269, 33.6%); (3) social phobia (n=193, 24.1%); and (4) specific phobia (n=70, 8.7%). The test set class categories were stratified to match the training set in proportion: 33.7% (90/267); 33.3% (89/267); 24% (64/267); and 9% (24/267) for the 4 classes, respectively.

The percentages of training or test set data per class were: 34% (269/801); 34% (269/801); 24% (193/801); and 9% (70/801) (rounded), respectively. Given this class distribution, we opted against upsampling the underrepresented classes (social phobia and specific phobia) or downsampling the overrepresented classes (depression and panic). While upsampling could mitigate class imbalance, it risks introducing synthetic patterns that may not generalize well to real-world data. In addition, given the relatively small sample size of the latter category (70/801, 9% in training), aggressive upsampling could lead to overfitting, where the model learns artifacts of the duplicated instances rather than underlying patterns in the data.

Conversely, downsampling depression and panic would reduce the total available training data and potentially discard valuable information, impairing the model’s ability to capture the full variation within these classes. Furthermore, since the dataset represents naturally occurring clinical distributions, artificial class balancing could distort the real-world relevance of model predictions. Instead, we opted to maintain the original class distribution and address class imbalance using performance metrics robust to class imbalance, such as balanced accuracy, weighted *F*_1_-score, and AUC, ensuring that model evaluation accounts for differences in class representation.

**Table 1:** Dataset features.

ID	Feature	Description	Feature type and values
1	Education	Years of education	Ordinal 0: primary school (0 to 9th grade) 1: high school (10th to 12th grade) 2: vocational education 3: short further education (≤3 years) 4: intermediate further education (4 or 5 years) 5: long further education (≥5 years) 6: other
2	AlcoholIntake	Weekly alcohol use (units)	Ordinal 0: 0 1: 1-5 or 1-7 2: 6-10 or 8-13 3: 11-20 or 14-20 4: 21-30 5: >30
3	UseOfDrugs	Use of euphoric drugs or pills	Categorical 0: no 1: yes
4	CurrentMedication	Current psychopharmacological treatment	Ordinal: 0: none 1: <1 month 2: 1 to 2 months 3: ≥2 months
5-13	Phq{One: Nine}	All 9 questions of PHQ-9^a^ [42] over the previous 2 weeks scored on a 4-point Likert scale	Ordinal 0: not at all 1: several days 2: more than half the days 3: nearly every day
14	PhqTen	Assessing to what extent the symptoms reported using PHQ-9 have affected work, household tasks, and relationships scored on a 4-point Likert scale	Ordinal 0: not difficult at all 1: somewhat difficult 2: very difficult 3: extremely difficult
15-21	Gad{One:Seven)	All 7 questions of GAD-7^b^ [43] scored on a 4-point Likert scale	Ordinal 0: not at all 1: several days 2: more than half the days 3: nearly every day
22	HasPhobia	Binary categorical variable of self-reported previous phobia diagnosis	Categorical 0: no previous phobia 1: previous phobia
23-46	Fq{One:TwentyFour}	All 24 questions of the Fear Questionnaire [46] Items 1-17 assess avoidance. Item 18 assesses phobic symptom severity. Items 19-24 assess how reported phobia affects the respondent.	Items 1-17: ordinal 0: would not avoid it 2: slightly avoid it 4: definitely avoid it 6: markedly avoid it 8: always avoid it Item 18: ordinal 0: no phobias present 2: slightly disturbing or not really disabling 4: definitely disturbing or disabling 6: markedly disturbing or disabling 8: very severely disturbing or disabling Items 19-24: ordinal 0: hardly at all 2: slightly troublesome 4: definitely troublesome 6: markedly troublesome 8: very severely troublesome
47-66	Sias{One:Twenty}	SIAS^c^ questionnaire [44] assesses the respondent’s social interaction anxiety using 20 statements, which the respondent indicates agreement with (“... characteristic or true of me”) on a 5-item Likert scale	Ordinal 0: not at all 1: slightly 2: moderately 3: very 4: extremely
67-73	Pdss{One:Seven}	PDSS^d^ questionnaire [45]	Separate ordinal answer key for each item with severity of responses rated as follows: 0: no or not 1: mild, occasional, or slight 2: moderate or significant 3: severe, very often, or substantial 4: extreme or nearly constantly
74,75,76	MansaTen, MansaTwentyFour, MansaTwentyFive	Questions 10, 24, and 25 of the 25-item MANSA^e^ questionnaire [47]	Ordinal 1: could not be worse 2: displeased 3: mostly dissatisfied 4: mixed 5: mostly satisfied 6: pleased 7: couldn’t be better
77-80	DAD	Previous diagnosis one-hot encoded using dummy variables DAD0-DAD3	One-hot encoded DAD0: not previously diagnosed with anxiety or depression DAD1: previously diagnosed with depression DAD2: previously diagnosed with anxiety DAD3: previously diagnosed with anxiety and depression
81-87	INC	Describing source of income of the respondent. One-hot encoded using dummy variables INC_1 to INC_7	One-hot encoded INC_1: employed INC_2: social security INC_3: sickness benefit or pay INC_4: unemployment benefit INC_5: stipend INC_6: pension INC_7: other
88-91	APP	Treatment applied for one-hot encoded using dummy variables APP_1 to APP_4	One-hot encoded APP_1: applied for anxiety treatment APP_2: applied for depression treatment APP_3: applied for both anxiety and depression treatment APP_4: does not know what treatment to apply for
GT	TreatmentType	Treatment decision (ground truth)	Categorical 1: depression 2: panic 3: social anxiety 4: specific phobia

^a^PHQ-9: Patient Health Questionnaire-9.

^b^GAD-7: Generalized Anxiety Disorder Questionnaire-7.

^c^SIAS: Social Interaction Anxiety Scale.

^d^PDSS: Panic Disorder Severity Scale.

^e^MANSA: Manchester Short Assessment of Quality of Life.

### Ethical Considerations

Data were extracted from Internetpsykiatrien after approval from The Regional Council in Southern Denmark. Since this was a secondary data analysis, separate informed consent was not required. However, the Regional Committees on Health Research Ethics for Southern Denmark were informed about the study and were provided with the case number S-20232000-65. In accordance with Danish national ethical guidelines, no additional ethics approval was needed. The study was reported to the Danish Data Protection Agency. Data was anonymized before being provided to the researchers. No compensation was provided.

### Model

The proposed model was designed to interpret the traditional sum-score questionnaires used in mental health treatment in a data-driven, explainable fashion. Sum-score questionnaires offer several prespecified answers to each question, with each answer associated with a numeric, typically integer, score. These scores are then summed and in many cases a threshold is then applied, which indicates whether the respondent demonstrates clinically significant symptoms for the disorders tested by the instrument. Hence, such sum-scores play an important role in clinical decision-making. ML models such as logistic regression strongly align with this principle while also offering inherent interpretability due to the nature of the output activation being related to a weighted sum of the inputs. Such inherent explainability is appealing; however, the complexity of the information conveyed when models contain many weights can lead to cognitive overload, hampering decision-making [15] and explainability [16].

**Figure 1:**
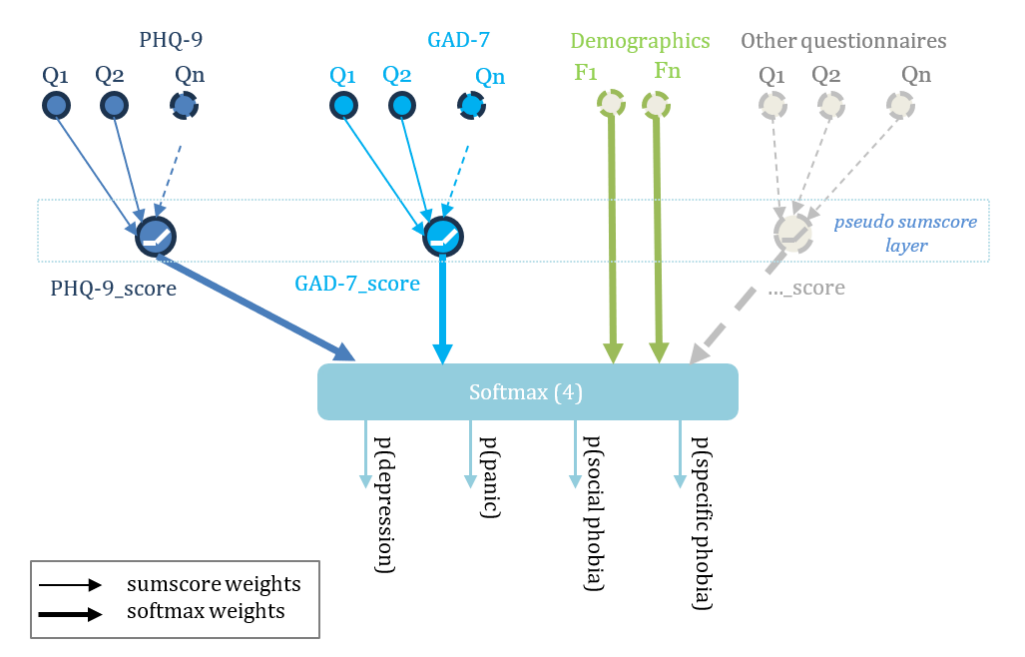
Model structure. The structure of the hierarchical model shows the questionnaire input features (Q) being passed to the pseudosum score layer and then to the output layer. The pseudosum layer incorporates a rectified linear unit activation function to ensure the pseudosum scores cannot be negative. The output layer performs a softmax function, predicting the probabilities of the 4 classes. The weights are represented by the arrows, with the thicker arrows representing the weights in the softmax layer. The demographic features (f) pass directly to the output layer with softmax weightings. GAD-7: Generalized Anxiety Disorder Questionnaire-7; PHQ-9: Patient Health Questionnaire-9.

To improve the model’s explainability while also retaining its inherent interpretability, a hierarchical model was proposed. As can be seen in Figure 1, this model mimics the sum-score models in the hidden layer of the model, in which the responses of each individual instrument are multiplied by their respective weight and then summed to yield pseudosum scores. These scores differ from normal sum-scores by applying a learned weighting to each answer before summing. These pseudosum scores incorporate a rectified linear unit activation function to ensure a linear sum while also ensuring the sum cannot be negative (due to the model learning negative weights during training). These scores and their weights allow inherent interpretability on a per-score basis, which will be familiar to clinical users of traditional questionnaire instruments. These pseudosum scores are then weighted before being combined in the output layer with the weighted sum-score of all other used instruments. In addition to the pseudosum scores, any other features with potentially discriminative power, such as sociodemographical information, can be added. All weights have dropout applied during prediction, consistent with the use of MCD as described previously. A softmax-type activation function is used in the output layer. The softmax activation function ensures that the sum of all the outputs of the model equals 1. Hence, each of the outputs can be interpreted as the probability that the corresponding treatment should be recommended. This output probability is illustrated as p(depression), as shown in Figure 1. Note that the output layer will typically contain 1 output node for each of the treatments considered by the model and that this number is not necessarily the same as the number of screening instruments used as inputs to the model.

With this specific architecture, the model achieved three important goals as follows: (1) the model allows the nonlinear combination of multiple instruments to achieve a treatment prediction, (2) the model allows the explanation of its predictions based on pseudosum score results and thus provides score-level explainability and trust in the model, and (3) at the same time, the model allows for the analysis of model predictions based on the input features, enabling users to understand which particular responses to each questionnaire drive the model predictions. These insights may provide therapists with valuable information on how to personalize the therapy.

The model’s structure inherited its interpretability from its foundation in logistic regression, while confidence and robustness of the model were addressed using the aforementioned MCD. As previously stated, MCD can be shown to approximate the probabilistic results obtained by Bayesian approaches for a class of models called Gaussian Processes. The latter are a generalization of generalized linear models, one of which is the logistic regression model chosen in our approach for its explainability. The hierarchical logistic regression model has dropout applied before every weight layer, since the Bayesian properties of MCD apply for neural networks of arbitrary depth and nonlinearities, as long as dropout is applied in this way [39]. While it is usual for dropout to be applied to neural networks to fulfill the function of regularization during training time [38], as explained, MCD also applies dropout during inference. The probability of a node being dropped, the dropout rate, is a parameter of the dropout layers. In MCD, the dropout rate is typically set heuristically. Extensions to the technique, such as learnable Bernoulli dropout [48], allows the dropout rate to be jointly optimized in the model training phase. When MCD is used in this way, to approximate a Bayesian network, it should not be seen as a regularization method [49]. Therefore, L1 regularization is applied during training to reduce overfitting. L1 regularization attempts to achieve good generalization (ie, good performance on yet unseen data) by forcing the influence of unimportant features to 0. L2 regularization can be applied as an alternative, fulfilling the regularization function without enforcing sparsity in the weights. Further justification for the choice of L1 regularization is provided in the Results section. Multimedia Appendix 1 shows the model details.

### Prediction Explainability and Confidence

**Figure 2:**
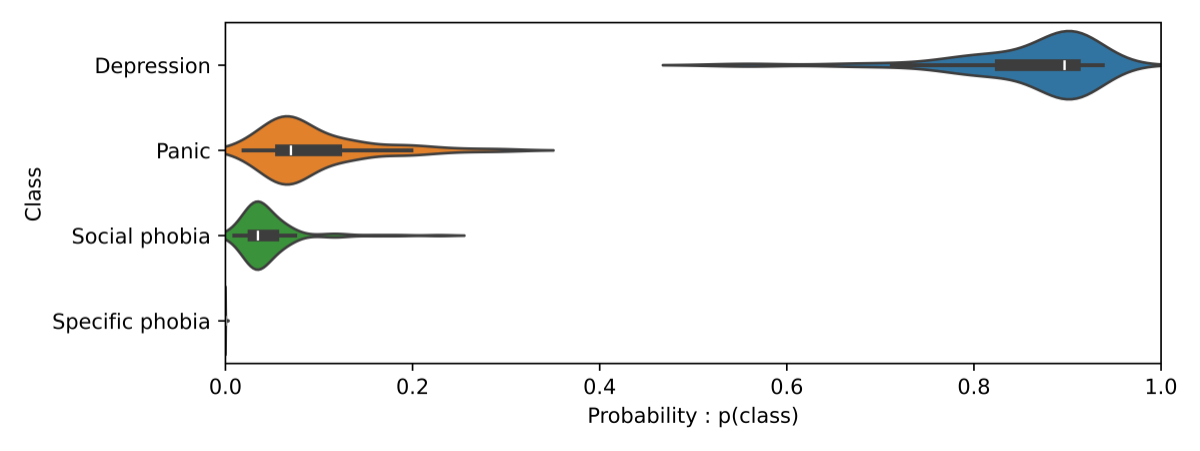
A representation of the output class distribution. A violin plot showing the probability distribution of the 4 treatment classes for a single example over N runs (samples) of Monte Carlo dropout. The separation of the probabilities (x-axis) indicates high model confidence in the prediction of depression compared to the other classes. The low dispersion in the predictions indicates low uncertainty.

**Figure 3:**
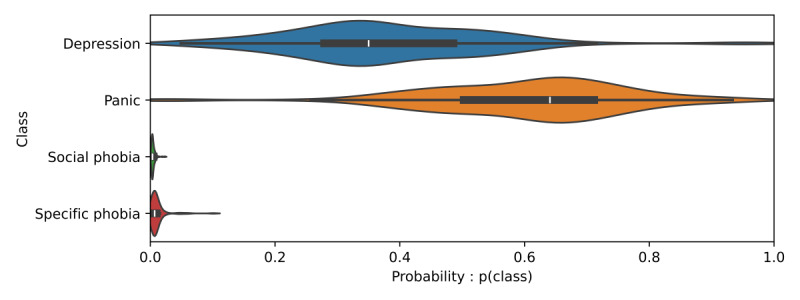
Class distribution of high uncertainty and low confidence. A violin plot showing the probability distribution of the 4 treatment classes for a single example over N runs (samples) of Monte Carlo dropout. The overlap of the probabilities (x-axis) indicates low model confidence in the prediction of panic compared to depression. The high dispersion in the predictions indicates high uncertainty.

The distribution of the softmax output classes over the N samples 
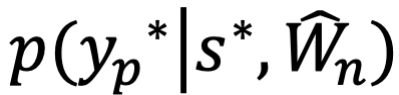
 , are visualized using Violin plots of the output class probability distribution over the N samples of a single patient prediction. A prediction for a single patient is shown in Figures 2 and 3. The outer, colored, “violin” shape visualizes the probability distributions over the N samples of the single patient example, while the embedded box-plots illustrate the medians and IQR of the model’s predictions. The confidence and uncertainty of the prediction can be understood by observing the shape and overlapping of the violin plots. Figure 2 provides an example of depression being predicted with high likelihood and nonoverlapping with the other 3 classes, whereas the predictions for all 3 other disorders have a low median probability. Predictions with low confidence (ie, low probabilities) and high uncertainty (ie, large probability variation), where more than 1 class may credibly be predicted, resulted in outputs, such as shown in Figure 3. This is an example of the relation between XAI concepts [16], using visualization to enhance explainability, contributing to improved interpretability, and the interpretability of the model leading to validation of the explanation.

### Prediction Interpretability and Robustness

Interpretability at the model level is aided by examining the learned weights after training. Since the scores are weighted linear sums of the question inputs W^T^X, the learned weights (W) are informative of the importance of each of the question features in determining the score used in the output class predictions in the softmax layer. The softmax layer implements a multiclass logistic regression function for each class (subscript c) and score (subscript s):



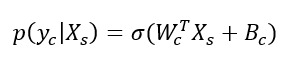



**(4)**

Where σ represents the sigmoid (or logistic) function, X_s_ is the input coming from the score layer or demographic features, W_c_ represents the weights in the softmax layer for class c, and B_c_ represents a bias value for each class.

Determining feature importance is an important aspect of explainability. At the model level, applying L1 regularization to logistic regression tends to force the weights of unimportant features to zero, leaving more important features as nonzero [50]. However, it is important to be aware that collinear features may not be equally important for a model since they can be accounted for by just one of the collinear features, with the others set to 0. Constraining trained weights to be nonnegative in the score layer aids interpretability since traditionally, questions are summed to produce a score. Therefore, the relative weights play a useful role in determining feature importance, provided that the inputs have been normalized to the same scale during training and prediction.

At the model level, standardized coefficients obtained by multiplying the original coefficients by the SD of the corresponding feature, provides a scale-free measure of influence, allowing the comparison of the relative importance of different features [51].

At the prediction level, for a specific patient record, the weights can be interpreted as the feature importance, given the normalized input range and positive weight constraint.

## Results

### Overview

The model was constructed using the TensorFlow library for Python (Python Software Foundation) and trained over 2000 epochs with a batch size of 32. The Adam optimizer was used at default setting, with L1 regularization of 0.001 and a dropout rate of 0.05 [52].

During development, both L1 and L2 regularization were considered, with evaluations involving 5-fold cross-validation on the training set. The optimal balanced accuracy score for both L1 and L2 corresponded to a regularization factor of 0.001 for both, with L1 and L2 achieving similar cross-validated mean balanced accuracy scores. Since L1 regularization tends to lead to a sparse solution by forcing unrequired weights to 0, it may be favored over L2 for transparent interpretability. However, when there are multiple near-equivalent predictors (eg, correlated measures), small fluctuations in initialization can lead the model to “select” a slightly different subset of features for its nonzero coefficients, eliminating one correlated feature, for example, while retaining the other. In consistency tests over 30 training runs, consistency in the top features was observed, although the order of importance may switch the top positive weight. In the depression class, for example, the top positive feature was PHQ-9 in 70% (21/30) of the runs and DAD_1.0 (previously diagnosed with depression) in 30% (9/30) of the runs. In the panic class, PDSS was always the top positive feature, with GAD-7 taking second place in 40% (12/30) of the runs and fq_total in 30% (9/30) of the runs. Examining the softmax weights, 80% (24/30) of the cumulative sum of the weights for each class were represented in the top 6 factors for L1 regularization, whereas for L2, up to 11 factors were required to represent the 80% (24/30). Therefore, L1 regularization was adopted for interpretability since: (1) both L1 and L2 regularization demonstrated similar accuracy; (2) L1 regularization enforces sparsity, requiring fewer weights to explain the top 80% (24/30); (3) the top weights are consistently in the top 3; and (4) the model transparency ensures the same weights are used in both prediction and explanation.

Following training, the model was evaluated on the held-out test set, achieving a balanced accuracy score of 0.79, indicating good generalization. The validation metrics (Table 2) indicate strong predictive performance across all 4 classes, with *F*_1_-scores ranging from 0.71 to 0.86, which indicates a balanced trade off between precision and recall. Specific phobia achieved the highest precision (0.84) and the highest recall (0.88), suggesting that the model is highly accurate in predicting this class when it does make a prediction. However, social phobia had the lowest recall (0.70) compared to other classes, indicating some difficulty in correctly identifying all cases of this condition. The AUC values, all >0.90, suggest strong discrimination between classes. The aggregated metrics demonstrate an overall balanced accuracy score of 0.79 and a weighted *F*_1_-score of 0.78, confirming that class imbalance did not severely impact overall model performance. The use of these weighted metrics ensures that smaller classes, such as specific phobia, contribute proportionally without distorting the results.

The confusion matrix (Table 3) details the number of treatment recommendations proposed by the model, with correct classifications appearing on the main diagonal of the table. Off-diagonal elements quantify the number of cases in which the model chose a different treatment than the clinician.

Patients assessed with social phobia were misclassified as depression in 4.9% (13/267) cases and as panic disorder in 1.9% (5/267) cases, suggesting that the model struggles to distinguish social phobia from other anxiety-related conditions. This was consistent with the lower recall observed for social phobia in Table 2, indicating that some patients with social phobia were incorrectly classified into other categories.

For specific phobia, the model correctly identified 7.9% (21/267) cases, but 1.1% (3/267) cases were misclassified as panic disorder (2/267, 0.7% cases) or social phobia (1/267, 0.4% case). Given that specific phobia was the least represented category in the dataset, the model’s relatively strong performance in this class may be influenced by its high specificity (0.98, Table 2), which suggests that when the model does predict specific phobia, it does so with a high degree of accuracy. However, the smaller sample size in this class may also lead to performance variability.

The model generally performed well in classifying depression and panic disorder, with high agreement between model predictions and clinician labels (72/267, 27% and 69/267, 25.8% correct classifications, respectively). However, 3.7% (10/267) panic disorder cases were misclassified as depression, indicating some overlap in how these conditions were represented in the feature space or possible comorbidities.

**Table 2:** Validation metrics on the test set.

Treatment	Precision	Recall (sensitivity)	*F*_1_-score	Specificity	Per-class balanced accuracy	AUC^a^
Depression	0.76	0.81	0.78	0.87	0.84	0.93
Panic	0.82	0.77	0.79	0.91	0.84	0.92
Social phobia	0.71	0.70	0.71	0.91	0.81	0.91
Specific phobia	0.84	0.88	0.86	0.98	0.93	0.98

^a^AUC: area under the curve.

**Table 3:** Confusion matrix on the test set.

Clinician proposed treatment (ground truth)	Model proposed treatment, n (%)			
	Depression	Panic disorder	Social phobia	Specific phobia
Depression	72 (27)	8 (3)	9 (3.4)	0 (0)
Panic	10 (3.7)	69 (25.8)	8 (3)	3 (1.1)
Social phobia	13 (4.9)	5 (1.9)	45 (16.8)	1 (0.4)
Specific phobia	0 (0)	2 (0.7)	1 (0.4)	21 (7.9)

### Model Level (Global) Explainability of Classes From Score Totals and Demographics

**Figure 4:**
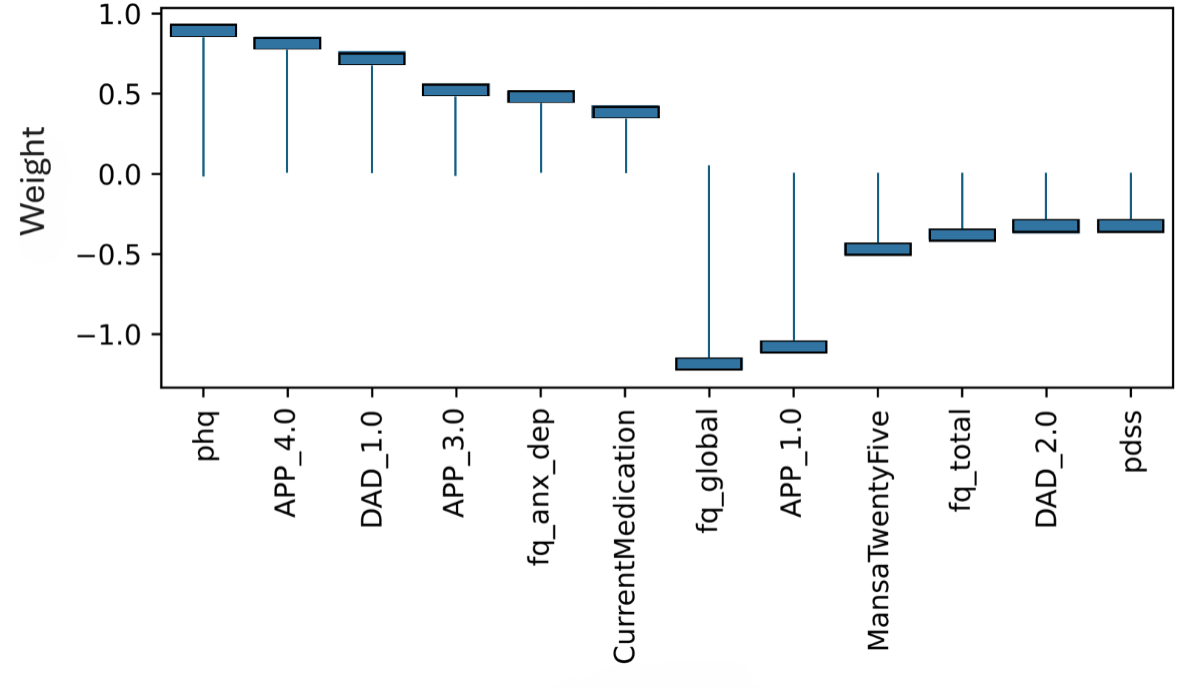
Weights of the softmax depression class node (top 6 positive and negative). The chart shows the top 6 positive and negative weights of the softmax output layer with respect to the depression class. The relative magnitude and sign indicate the importance of the feature for the prediction. Positive weights contribute to the class prediction probability. Negative weights detract from it. The chart indicates that the Patient Health Questionnaire (PHQ-9) score (phq), and application-related variables (APP_4.0, APP_3.0) have the most influence on the prediction of the depression class, while the Fear Questionnaire Global Phobia rating (fq_global) has the strongest negative influence.

**Figure 5:**
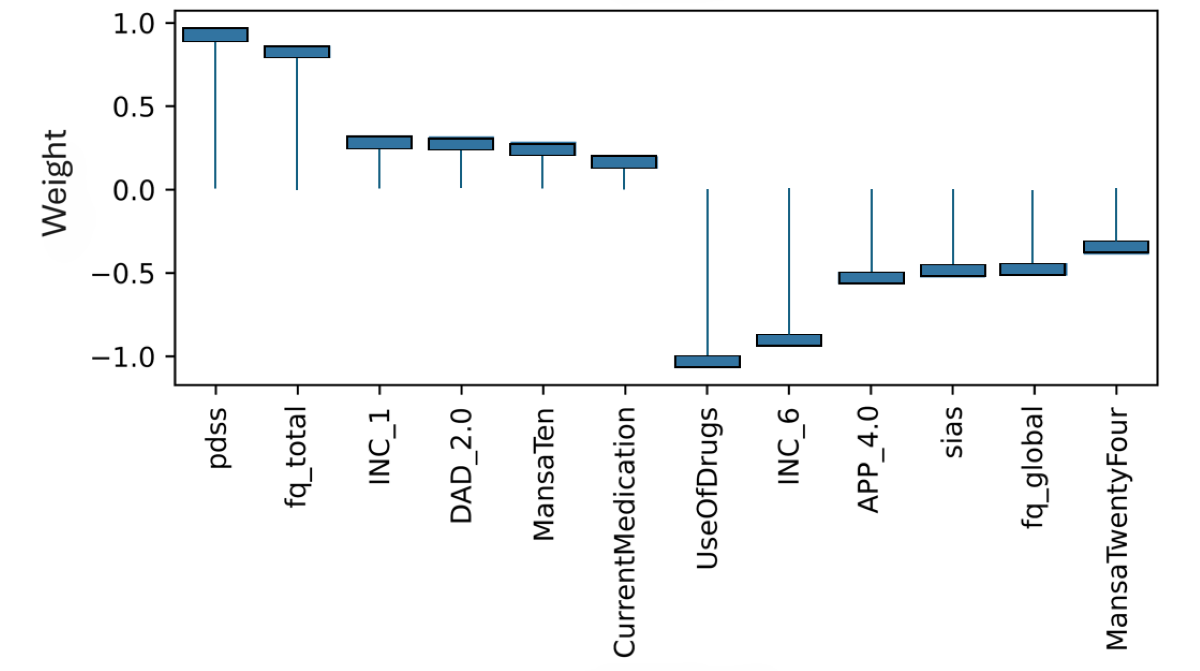
Weights of the softmax panic class node (top 6 positive and negative). The chart shows the top 6 positive and negative weights of the softmax output layer with respect to the panic class. The relative magnitude and sign indicate the importance of the feature for the prediction. Positive weights contribute to the class prediction probability. Negative weights detract from it. The chart indicates that the Panic Disorder Severity Scale (PDSS) score (pdss), and the fear questionnaire total score (fq_total) have the most influence on the prediction of the panic class, while the use of euphoric drugs or pills (UseOfDrugs) has the strongest negative influence.

**Figure 6:**
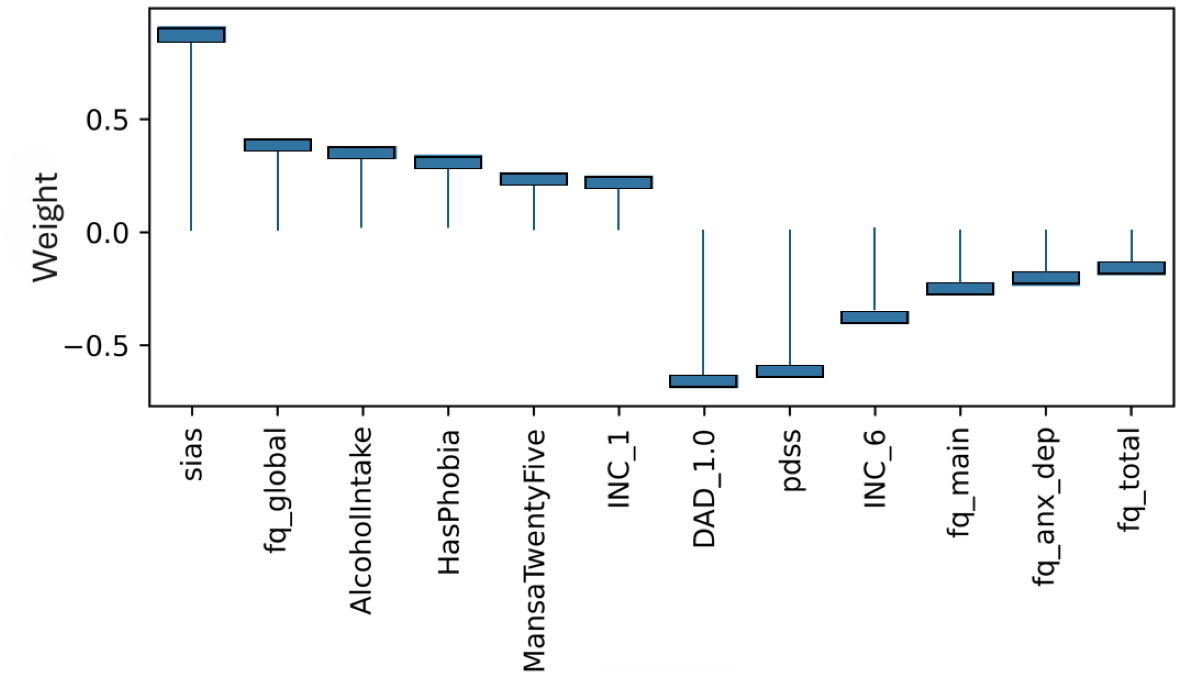
Weights of the softmax social phobia class node (top 6 positive and negative). The chart shows the top 6 positive and negative weights of the softmax output layer with respect to the social phobia class. The relative magnitude and sign indicate the importance of the feature for the prediction. Positive weights contribute to the class prediction probability. Negative weights detract from it. The chart indicates that the SIAS score (sias) and the Fear Questionnaire Global Phobia rating (fq_global) have the most influence on the prediction of the social phobia class. Having been previously diagnosed with depression (DAD_1.0) has the strongest negative influence.

**Figure 7:**
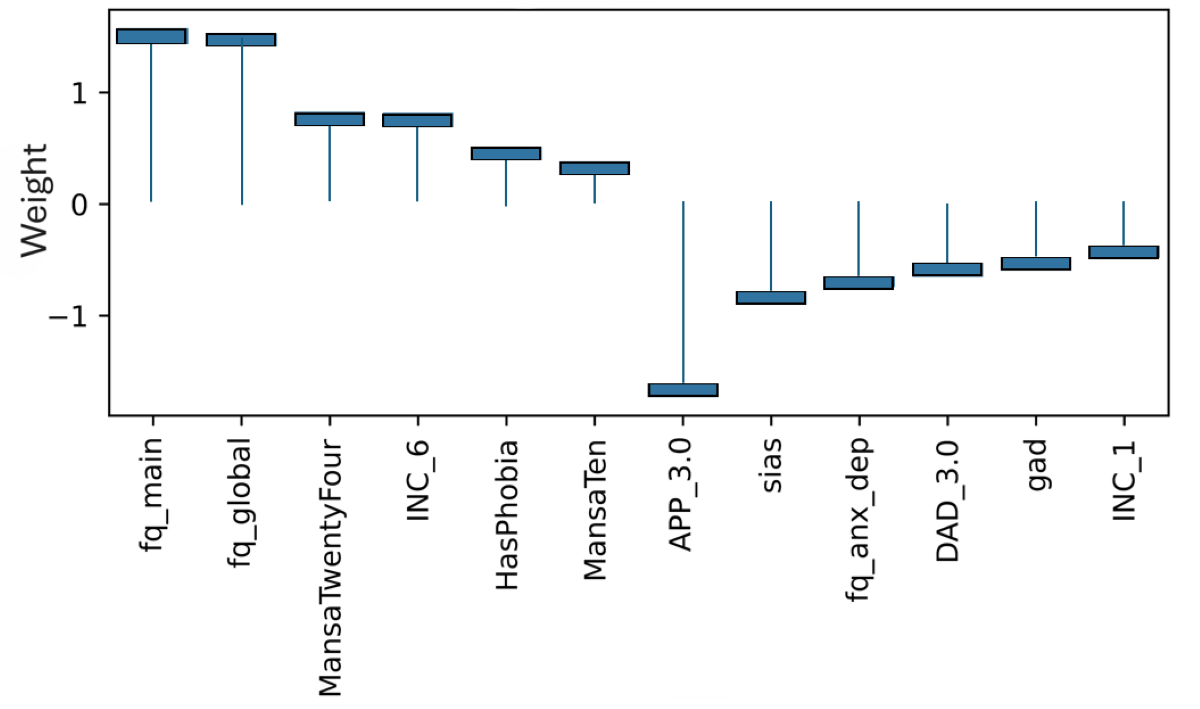
Weights of the softmax specific phobia class node (top 6 positive and negative). The chart shows the top 6 positive and negative weights of the softmax output layer with respect to the specific phobia class. The relative magnitude and sign indicate the importance of the feature for the prediction. Positive weights contribute to the class prediction probability. Negative weights detract from it. The chart indicates that the Fear Questionnaire has the most influence on the prediction of the panic class. The main Phobia Level of Avoidance score (fq_main), and Global Phobia rating (fq_global) are the top 2 positive weights. Having applied for both anxiety and depression treatment, (APP_3.0) has the strongest negative influence.

Examining the learned weights of the softmax layer that predicts the class probabilities from the score and demographic features is informative to interpret their relative importance at the model level. Positive weights contribute toward a more probable class prediction, while negative weights detract. Examining the top 6 positive and negative contributions, the depression class (Figure 4), is mostly influenced by the PHQ-9 score, application-related variables (APP_4.0, APP_3.0), and the previous diagnosis of depression (DAD_1.0). The Fear Questionnaire Global Phobia rating (question 18) detracts from this class prediction. The panic class (Figure 5) is most influenced positively by the PDSS score and the Fear Questionnaire total score (fq_total) and most negatively by the use of euphoric drugs or pills (UseOfDrugs), so drug use detracts from this class probability. The social phobia class (Figure 6) is most influenced by the SIAS score and the Fear Questionnaire Global Phobia rating (fq_global). Having been previously diagnosed with depression (DAD_1.0) works against this class. The specific phobia class (Figure 7) is most influenced by the Fear Questionnaire main level of avoidance (item 1), and the Global Phobia rating (fq_global), while having applied for both anxiety and depression treatment (APP_3.0) counts against this class.

### Model Level (Global) Explainability of Score Totals

**Figure 8:**
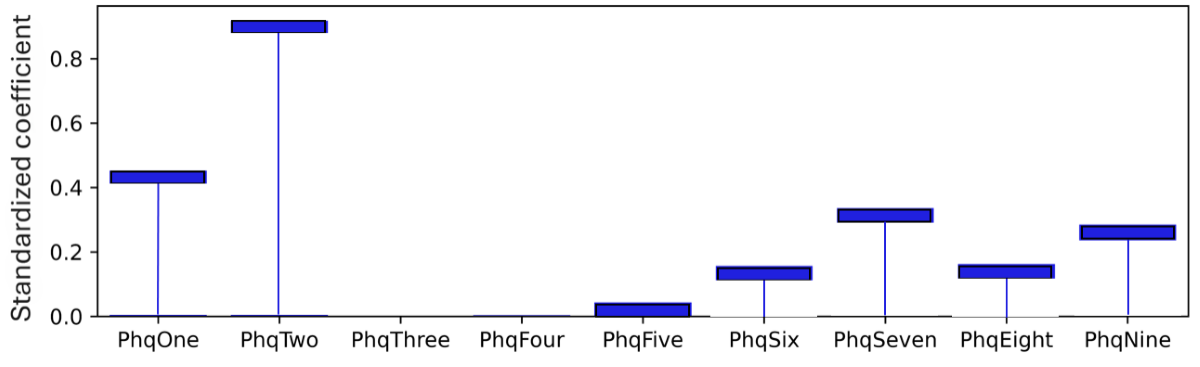
Patient Health Questionnaire-9 (PHQ-9) question standardized coefficients. This chart illustrates the relative importance of the PHQ-9 questions with respect to the pseudosum score for the PHQ-9 (phq) after model training.

**Figure 9:**
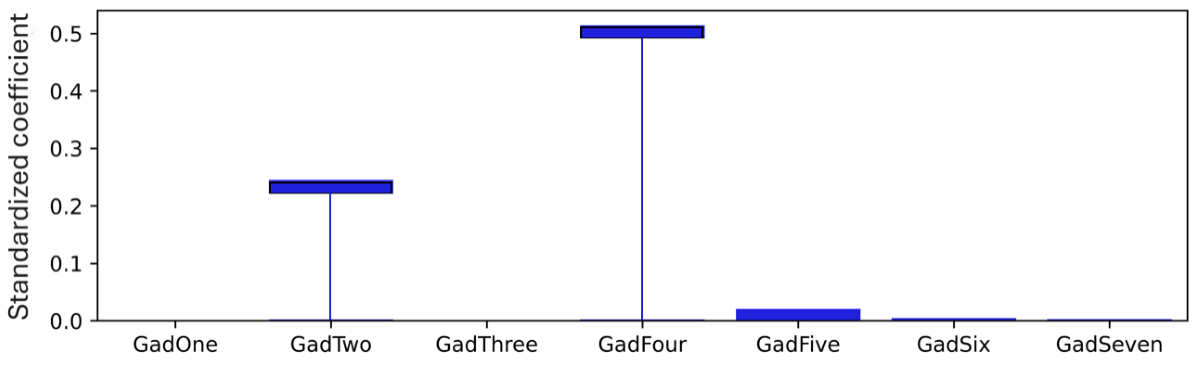
Generalized Anxiety Disorder Questionnaire-7 (GAD-7) question standardized coefficients. This chart illustrates the relative importance of the GAD-7 questions with respect to the pseudosum score for the GAD-7 (gad) after model training. GAD question 4 “Trouble relaxing” and GAD question 2 “Not being able to stop or control worrying,” are the main nonzero standardized coefficient after L1 regularization pushes unimportant weights to zero.

**Figure 10:**
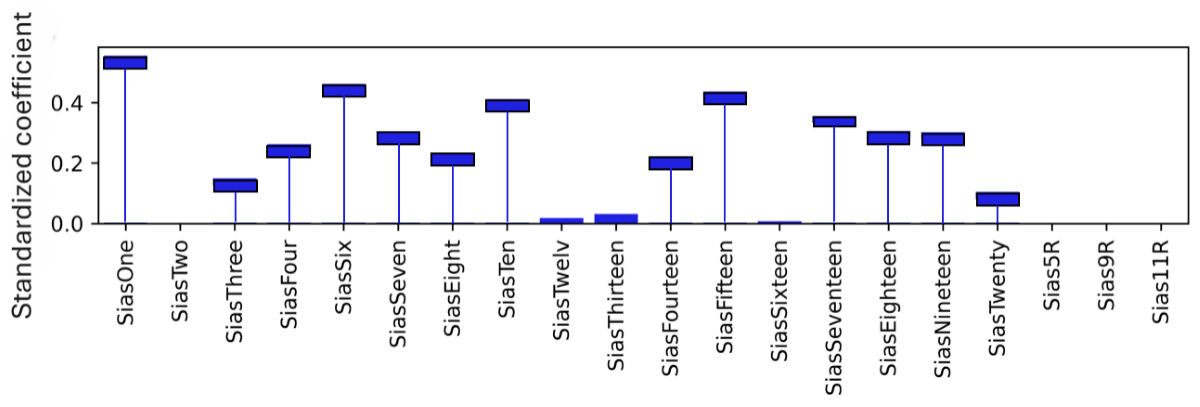
Social Interaction Anxiety Scale (SIAS) question standardized coefficients. This chart illustrates the relative importance of the SIAS questions with respect to the pseudosum score for the SIAS (sias) after model training. Reverse scored features have an R suffix, for example, Sias5R.

**Figure 11:**
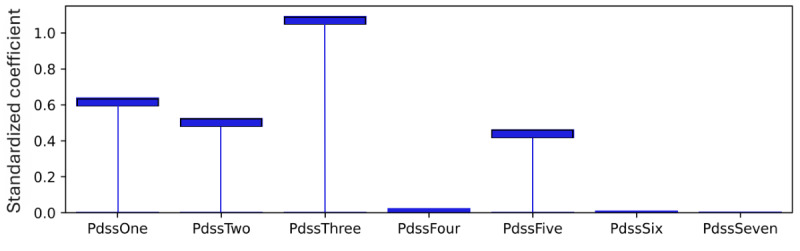
PDSS question standardised coefficients. This chart illustrates the relative importance of the PDSS questions with respect to the pseudo-sum score for the PDSS (pdss) after model training.

**Figure 12:**
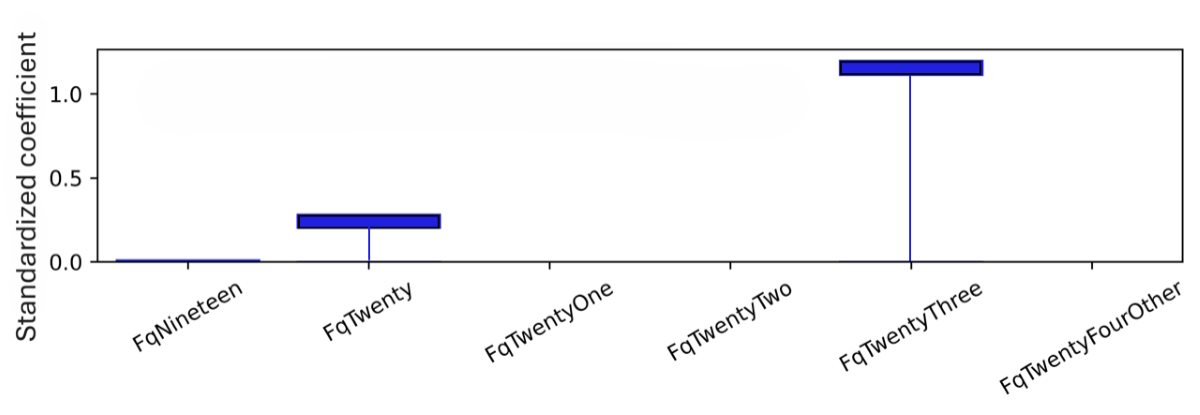
Fear Questionnaire (FQ) anxiety depression subscale question standardized coefficients. This chart illustrates the relative importance of the Fear Questionnaire Total Phobia Score questions with respect to the pseudosum score (fq) after model training. Question 23, “Feeling you or your surroundings are strange or unreal.” is the largest standardized coefficient after L1 regularization pushes unimportant weights to 0.

**Figure 13:**
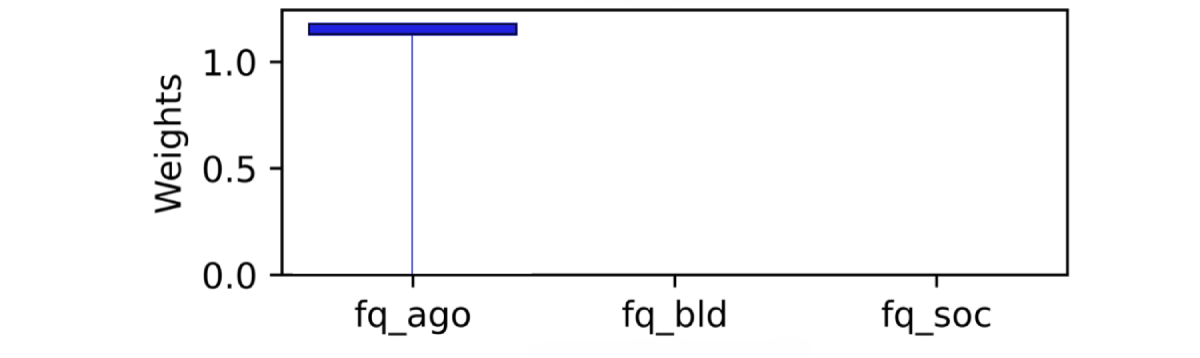
Fear Questionnaire (FQ) total score weighting of the FQ subscales. This chart illustrates how the Fear Questionnaire subscales are weighted in relation to the fear questionnaire total score fq_total. Only the agoraphobia subscale (fq_ago) was learned as being relevant. This is likely to be due to L1 regularization of correlated factors.

**Figure 14:**
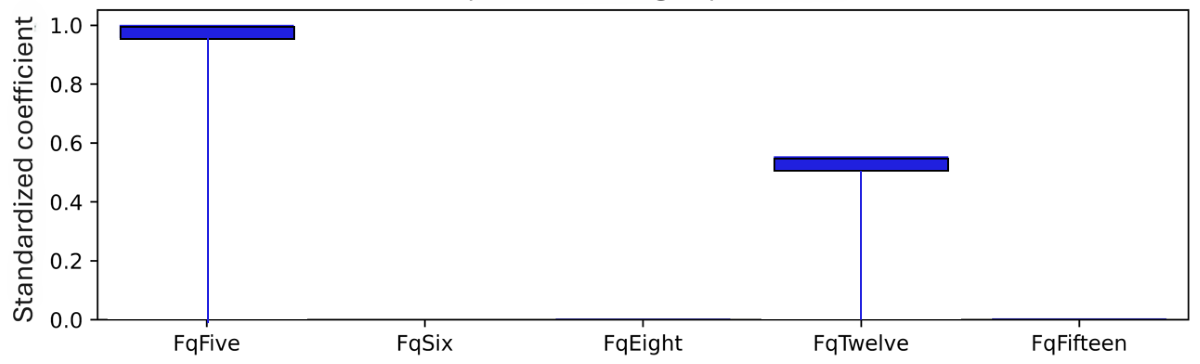
Fear Questionnaire (FQ) agoraphobia subscale question standardized coefficients. This chart illustrates the relative importance of the FQ agoraphobia subscore questions.

As explained, standardized coefficients obtained by multiplying the original coefficients by the SD of the corresponding feature in the dataset provide a scale-free measure of influence, allowing the comparison of the relative importance of different features. The standardized coefficients are shown in Figures 8-12. Examining the PHQ-9 standardized coefficients (Figure 8), questions 1 and 2, the main cognitive factors of the questionnaire [53] are the most important, with all the 5 cognitive factors being represented. The importance of the GAD-7 (Figure 9) is almost exclusively represented by questions 4 (“Trouble relaxing”) and 2 (“Not being able to stop or control worrying”). The most important SIAS questions (Figure 10) were 6 and 1. Question 3, (“How often do you feel anxious about the possibility of having a panic attack?”) was the most important in the PDSS (Figure 11). In the Fear Questionnaire anxiety or depression subscale (Figure 12), question 23, “Feeling you or your surroundings are strange or unreal,” was the most important. The Total Phobia Score from the Fear Questionnaire was defined as the sum of the subscale scores for agoraphobias, blood injury phobia, and social phobia. In this case, only the learned agoraphobia subscore was weighted at nonzero (Figure 13). Question 5 was the most relevant question in the Fear Questionnaire agoraphobia subscale (Figure 14).

### Patient Level (Local) Explainability

The relevant questionnaire total score summaries of a test patient (patient 1) are provided here. The PHQ-9 (15) score indicated moderately severe depression [42]. The PDSS score (13) indicated “moderately ill” panic disorder [54]. The GAD-7 score (17) indicated severe anxiety [55]. The SIAS score (22) was lower than the threshold for an indication of social phobia [56]. The clinical assessment (ground truth) of an appropriate treatment was panic. Other scores included Fear Questionnaire total score (6), global score (0), and main score (0).

**Figure 15:**
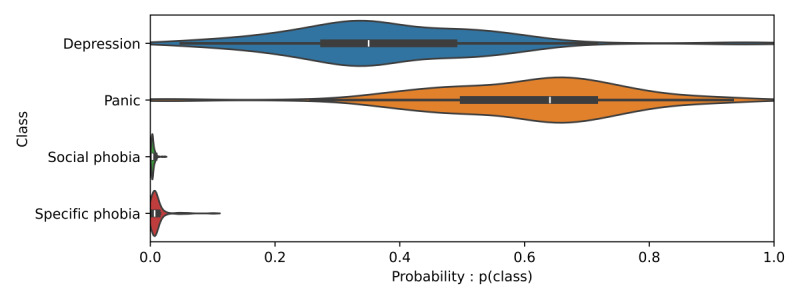
Prediction for patient 1. A violin plot showing the probability distribution of the 4 treatment classes for the data of patient 1. Although panic has the highest probability, the overlap of the probabilities (x-axis) indicates low model confidence in the prediction of panic compared to depression. The high dispersion in the predictions indicate high uncertainty. Overall, this plot indicates to a clinical user that both panic and depression are credible treatments according to the model.

**Figure 16:**
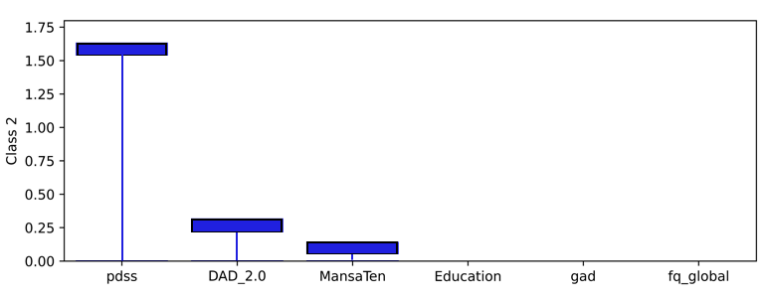
Patient 1 explainability for panic prediction (positive weightings). The product of the weights and the pseudosum score data or demographic data explains the importance of those features in determining the local prediction from a patient record. These positive values contribute to the class probability. The Panic Disorder Severity Scale (PDSS) score (pdss) is the most significant contributor to the probability of the panic class for patient 1.

**Figure 17:**
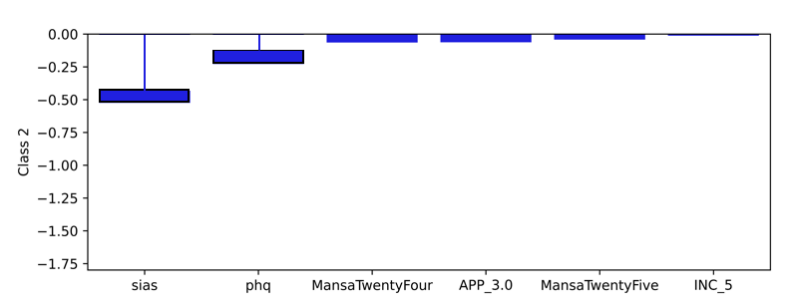
Patient 1 explainability for panic (class 2) prediction (negative weightings). The product of the weights and the pseudosum score data or demographic data explains the importance of those features in determining the local prediction from a patient record. These negative values detract from the class probability. The Social Interaction Anxiety Scale (SIAS) score (sias) is the most significant detractor from the probability of the panic class for patient 1, but at approximately −0.5, it has less contribution than the comparative positive scores.

Examining a local explanation at the individual patient level for patient 1 (Figure 15), the predicted probability distribution of panic (the prescribed treatment), overlapped with depression. The importance was determined from the weighted inputs to the softmax layer, combining both the patient record and the learned weights. Examining the importance associated with the prediction in the softmax layer for the panic class shows the top 6 positive factors (Figure 16), and the top 6 negative factors (Figure 17). Positive values make the class more probable, while negative values make the class less probable. It should be noted that the positive and negative y-values for each class are derived from the same weight layer and, therefore, are suitable for comparative analysis. In the patient 1 example, the PDSS questionnaire score explained most of the probability for the panic class, while the SIAS questionnaire detracted from the probability of the panic class, but to a lesser degree.

**Figure 18:**
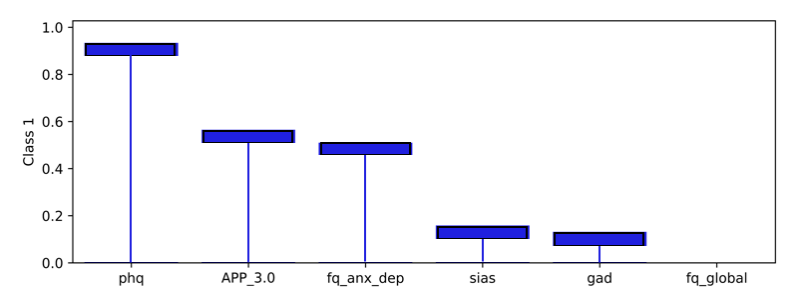
Patient 1 explainability for depression prediction (positive weightings). The product of the weights and the pseudosum score data or demographic data explains the importance of those features in determining the local prediction from a patient record. These positive values contribute to the class probability. The Patient Health Questionnaire-9 (PHQ-9) score (phq) is the most significant contributor to the probability of the depression class for patient 1.

**Figure 19:**
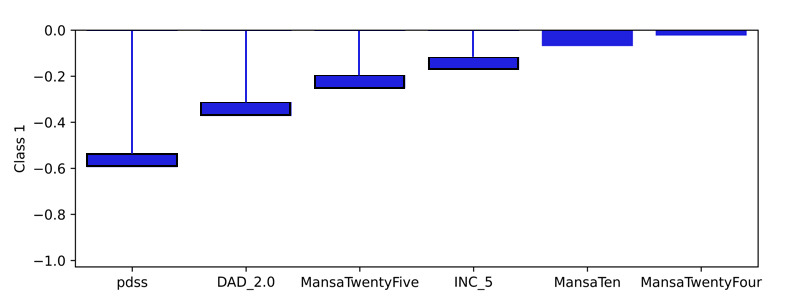
Patient 1 explainability for depression prediction (negative weightings). The product of the weights and the pseudosum score data or demographic data explains the importance of those features in determining the local prediction from a patient record. These negative values detract from the class probability. The Panic Disorder Severity Scale (PDSS) score (pdss) is the most significant detractor from the probability of the depression class for patient 1.

The model can also be used to explain any of the class probabilities. It is not limited to explaining the highest probability prediction. Hence, the class prediction for depression can be examined in the same way. This showed the PHQ-9 score as the most important (Figure 18), followed by APP_3 (the patient applied for both anxiety and depression treatment). The PDSS score showed a negative influence on this class (Figure 19), which was important but lower than the positive importance of PHQ-9. It is interesting to note that the negative importances for the depression class (Figure 19) were larger than the negative importances for panic, the highest probability class (Figure 17). This goes some way to explain why panic was predicted to be more probable than depression.

**Figure 20:**
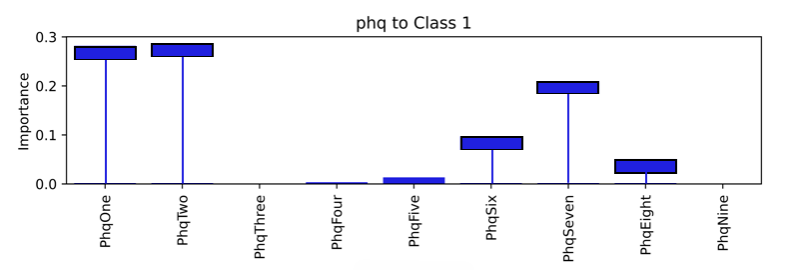
Patient 1 explainability for the Patient Health Questionnaire-9 (PHQ-9) questions in the depression prediction. The product of the weights and the question data explains the importance of the questions in determining the local prediction from a patient record. The PHQ-9 questions 1 and 2 score are the most significant contributors to the phq pseudosum score of depression class for patient.

The hierarchical model also allowed a closer examination of the individual questions. For example, an examination of the PHQ-9 questions that contributed to the probability of the depression class (Figure 20), showed PHQ-9 questions 1 and 2 as having the most influence on the model prediction for this class, for this patient.

## Discussion

### Overview

Uncertainty and lack of confidence in diagnosing psychopathological conditions can present a considerable challenge due to comorbidities and overlapping symptoms [6]. The diagnosis of social anxiety disorder, for example, must be differentiated from major depression, panic disorder, agoraphobia, and generalized anxiety disorder [7,57]. Furthermore, maintaining human involvement in patient assessments involving AI is critical [58]. Therefore, explaining predictions and enabling clinicians to explore the clinical factors associated with the predictions, has been a central aspect of this work.

### Principal Findings

We have demonstrated the design and quantitative evaluation of a hierarchical model that aims to aid diagnosis and meet the requirements of providing explainable predictions that are context-based and easy for clinicians to use in questionnaire-based assessments [4]. Issues of confidence and robustness that are important for ML models aimed at medical DSSs [25] have been addressed.

As the model achieved a balanced accuracy score of 0.79 and AUC >0.90, it is expected that it will be clinically useful in both screening a new patient and informing a clinical interview. In patient screening, for example, the graphical interpretation of the class probabilities, their spread, and their overlap can inform the clinician of competing treatment possibilities for the patient. Even in the case where the treatment prediction is unambiguous and agrees with the clinician’s assessment, the model may serve as a confirmatory second opinion. At the clinical interview stage, the specific questions that are deemed important for the class prediction may inform the clinician of which areas to probe further in the clinical interview.

### Limitations

ML models extract knowledge from data, limiting their applicability to use in scenarios that are statistically similar to the training scenario. The models reported in this study were trained on data obtained from the Internetpsykiatrien web-based treatment with nationwide coverage in Denmark [41]. Hence, while similar performance for individuals presenting to this service in Denmark can be expected, performance in a different country or otherwise different setting cannot be guaranteed. This can be seen as a limitation of ML models, but also as a strength; retraining the model described in this study is cheap. Hence, it is relatively straightforward for any regional mental health service to train this model on local data and obtain predictions that are sensitive to regional sociodemographics and patient characteristics. Moreover, patient characteristics may well change over time, and such temporal changes can be addressed by retraining the model periodically.

The ground truth is based on clinical judgment and is, therefore, influenced by the subjectivity of the clinicians, interrater variability, and possibly by group dynamics in decision-making clinical conferences. Clinicians have access to free-text provided by the patient, which the model does not have. A future direction may involve the development of a hybrid model that makes use of all the relevant questionnaire and demographic and free-text data for each patient. An additional limitation of the ground truth is that the assessment relies on structured questionnaires and the Mini Neuropsychiatric Interview, which may not capture all relevant patient characteristics, leading to potential gaps in the ground truth.

During training, L1 regularization adjusts the standardized coefficients and model weights toward 0 for features deemed unimportant or redundant, for example, due to having a high correlation with another feature. Consequently, features that clinicians consider relevant may be excluded by the model and thus do not appear in the explanations of predictions. However, it is common for clinicians to prioritize the importance of certain features over others in a diagnostic context [59]. It is anticipated that clinicians will find the specificity of the explanations at the question level to be clarifying and informative, complementing their own clinical judgment. Moreover, L1 regularization may result in model instability in the sense that small changes in the training set may result in a different selection of nonzero coefficients (and thus important features). Hence, a retrained model may yield different explanations to the original model for similar individuals, and this could be confusing to clinicians. However, it should be emphasized that the feature weights that explain the predictions are the same feature weights that the model uses to make the predictions, due to the model’s transparency. However, using L1 regularization is not a prerequisite for the use of the proposed approach. L2 regularization, which attempts to achieve good generalization by keeping model weights small (rather than forcing a subset to 0 such as in L1 regularization) can also be used. The result would be that the model contains mostly nonzero weights, and thus, the explainability of model decisions becomes more complex. However, clinicians could choose to focus on the most important features (ie, those with the highest influence on the model). Visualizations, such as those in Figures 16-19, could be constrained to the N most important features with N chosen by the clinician.

The clinical use of the ML model would require the development of a DSS so that clinicians can view and explore the model predictions and explanations when provided with patient records. The lack of such a DSS may be seen as a limitation in demonstrating the clinical effectiveness of the model. While this study does not empirically demonstrate that the aims of clinical interpretability have been met, further work is planned to develop the DSS and study the clinical usefulness of the system incorporating the model.

Overall, the results highlight the key challenges in differentiating social phobia from other anxiety disorders and suggests that future improvements could focus on enhancing feature differentiation for overlapping categories, particularly social phobia and panic disorder.

### Future Work

The incorporation of this model into a DSS for mental health treatment prediction will be an important future step for this work. Once developed, assessing its use in a clinical setting will help to validate it for future clinical use. The model itself could be extended to use more input features, especially those that have a potential to enhance feature differentiation for overlapping categories (particularly social phobia and panic disorder). Additional hybrid features may include free-text data input for each patient. Output enhancements can also be foreseen to further build on the model’s explainability. For example, a text output of the model’s prediction, competing predictions, and explanations in written form could form an important input to clinical notes and provide material for clinical conferences.

### Conclusions

This paper presents the design of a model aimed at a DSS for psychopathological mental health treatment. Its basis in standard screening questionnaires will allow seamless adoption in a clinical setting in the future. The AI model incorporated in the system is based on a novel hierarchical structure that aims to meet the needs of clinical interpretability while accounting for decision confidence and uncertainty that are lacking in previous work.

## Appendix

Multimedia Appendix 1 Details of the model layers and structure.

## Abbreviations

AI: artificial intelligence
AUC: area under the curve
DSS: decision support system
GAD-7: Generalized Anxiety Disorder Questionnaire-7
LIME: local interpretable model-agnostic explanations
MCD: Monte Carlo dropout
ML: machine learning
PDSS: Panic Disorder Severity Scale
PHQ-9: Patient Health Questionnaire-9
SHAP: Shapley additive explanations
SIAS: Social Interaction Anxiety Scale
XAI: explainable artificial intelligence
