# Supplementary material for: An Interpretable Model With Probabilistic Integrated Scoring for Mental Health Treatment Prediction: Design Study
Source: JMIR Med Inform. 2025 Mar 26;13:e64617. doi: 10.2196/64617 (PMC11982765; doi:10.2196/64617)
Supplement: Multimedia Appendix 1 [file medinform_v13i1e64617_app1.docx]

## Model Layer Summary

━━━━━━━━━━━━━━━━━━━━━┳━━━━━━━━━━━━━━━━━━━┳━━━━━━━━━━━━┳━━━━━━━━━━━━━━━━━━━┓

┃ **Layer (type)** ┃ **Output Shape** ┃ **Param #** ┃ **Connected to** ┃

┡━━━━━━━━━━━━━━━━━━━━━╇━━━━━━━━━━━━━━━━━━━╇━━━━━━━━━━━━╇━━━━━━━━━━━━━━━━━━━┩

│ input_layer_120 │ (None, 91) │ 0 │ - │

│ (InputLayer) │ │ │ │

├─────────────────────┼───────────────────┼────────────┼───────────────────┤

│ X_fq_ago │ (None, 5) │ 0 │ input_layer_120[… │

│ (GatherLayer) │ │ │ │

├─────────────────────┼───────────────────┼────────────┼───────────────────┤

│ X_fq_bld │ (None, 5) │ 0 │ input_layer_120[… │

│ (GatherLayer) │ │ │ │

├─────────────────────┼───────────────────┼────────────┼───────────────────┤

│ X_fq_soc │ (None, 5) │ 0 │ input_layer_120[… │

│ (GatherLayer) │ │ │ │

├─────────────────────┼───────────────────┼────────────┼───────────────────┤

│ D_fq_ago (Dropout) │ (None, 5) │ 0 │ X_fq_ago[0][0] │

├─────────────────────┼───────────────────┼────────────┼───────────────────┤

│ D_fq_bld (Dropout) │ (None, 5) │ 0 │ X_fq_bld[0][0] │

├─────────────────────┼───────────────────┼────────────┼───────────────────┤

│ D_fq_soc (Dropout) │ (None, 5) │ 0 │ X_fq_soc[0][0] │

├─────────────────────┼───────────────────┼────────────┼───────────────────┤

│ L_fq_ago (Dense) │ (None, 1) │ 5 │ D_fq_ago[0][0] │

├─────────────────────┼───────────────────┼────────────┼───────────────────┤

│ L_fq_bld (Dense) │ (None, 1) │ 5 │ D_fq_bld[0][0] │

├─────────────────────┼───────────────────┼────────────┼───────────────────┤

│ L_fq_soc (Dense) │ (None, 1) │ 5 │ D_fq_soc[0][0] │

├─────────────────────┼───────────────────┼────────────┼───────────────────┤

│ X_phq (GatherLayer) │ (None, 9) │ 0 │ input_layer_120[… │

├─────────────────────┼───────────────────┼────────────┼───────────────────┤

│ X_gad (GatherLayer) │ (None, 7) │ 0 │ input_layer_120[… │

├─────────────────────┼───────────────────┼────────────┼───────────────────┤

│ X_sias │ (None, 20) │ 0 │ input_layer_120[… │

│ (GatherLayer) │ │ │ │

├─────────────────────┼───────────────────┼────────────┼───────────────────┤

│ X_pdss │ (None, 7) │ 0 │ input_layer_120[… │

│ (GatherLayer) │ │ │ │

├─────────────────────┼───────────────────┼────────────┼───────────────────┤

│ gather_layer_360 │ (None, 6) │ 0 │ input_layer_120[… │

│ (GatherLayer) │ │ │ │

├─────────────────────┼───────────────────┼────────────┼───────────────────┤

│ concatenate_240 │ (None, 3) │ 0 │ L_fq_ago[0][0], │

│ (Concatenate) │ │ │ L_fq_bld[0][0], │

│ │ │ │ L_fq_soc[0][0] │

├─────────────────────┼───────────────────┼────────────┼───────────────────┤

│ D_phq (Dropout) │ (None, 9) │ 0 │ X_phq[0][0] │

├─────────────────────┼───────────────────┼────────────┼───────────────────┤

│ D_gad (Dropout) │ (None, 7) │ 0 │ X_gad[0][0] │

├─────────────────────┼───────────────────┼────────────┼───────────────────┤

│ D_sias (Dropout) │ (None, 20) │ 0 │ X_sias[0][0] │

├─────────────────────┼───────────────────┼────────────┼───────────────────┤

│ D_pdss (Dropout) │ (None, 7) │ 0 │ X_pdss[0][0] │

├─────────────────────┼───────────────────┼────────────┼───────────────────┤

│ D_fq_anx_dep │ (None, 6) │ 0 │ gather_layer_360… │

│ (Dropout) │ │ │ │

├─────────────────────┼───────────────────┼────────────┼───────────────────┤

│ D_fq_total │ (None, 3) │ 0 │ concatenate_240[… │

│ (Dropout) │ │ │ │

├─────────────────────┼───────────────────┼────────────┼───────────────────┤

│ L_phq (Dense) │ (None, 1) │ 9 │ D_phq[0][0] │

├─────────────────────┼───────────────────┼────────────┼───────────────────┤

│ L_gad (Dense) │ (None, 1) │ 7 │ D_gad[0][0] │

├─────────────────────┼───────────────────┼────────────┼───────────────────┤

│ L_sias (Dense) │ (None, 1) │ 20 │ D_sias[0][0] │

├─────────────────────┼───────────────────┼────────────┼───────────────────┤

│ L_pdss (Dense) │ (None, 1) │ 7 │ D_pdss[0][0] │

├─────────────────────┼───────────────────┼────────────┼───────────────────┤

│ L_fq_anx_dep │ (None, 1) │ 6 │ D_fq_anx_dep[0][… │

│ (Dense) │ │ │ │

├─────────────────────┼───────────────────┼────────────┼───────────────────┤

│ L_fq_total (Dense) │ (None, 1) │ 3 │ D_fq_total[0][0] │

├─────────────────────┼───────────────────┼────────────┼───────────────────┤

│ gather_layer_361 │ (None, 1) │ 0 │ input_layer_120[… │

│ (GatherLayer) │ │ │ │

├─────────────────────┼───────────────────┼────────────┼───────────────────┤

│ gather_layer_362 │ (None, 1) │ 0 │ input_layer_120[… │

│ (GatherLayer) │ │ │ │

├─────────────────────┼───────────────────┼────────────┼───────────────────┤

│ X_other │ (None, 23) │ 0 │ input_layer_120[… │

│ (GatherLayer) │ │ │ │

├─────────────────────┼───────────────────┼────────────┼───────────────────┤

│ concatenate_241 │ (None, 31) │ 0 │ L_phq[0][0], │

│ (Concatenate) │ │ │ L_gad[0][0], │

│ │ │ │ L_sias[0][0], │

│ │ │ │ L_pdss[0][0], │

│ │ │ │ L_fq_anx_dep[0][… │

│ │ │ │ L_fq_total[0][0], │

│ │ │ │ gather_layer_361… │

│ │ │ │ gather_layer_362… │

│ │ │ │ X_other[0][0] │

├─────────────────────┼───────────────────┼────────────┼───────────────────┤

│ dropout_120 │ (None, 31) │ 0 │ concatenate_241[… │

│ (Dropout) │ │ │ │

├─────────────────────┼───────────────────┼────────────┼───────────────────┤

│ Softmax (Dense) │ (None, 4) │ 128 │ dropout_120[0][0] │

└─────────────────────┴───────────────────┴────────────┴───────────────────┘

**Total params:** 195 (780.00 B)

**Trainable params:** 195 (780.00 B)

**Non-trainable params:** 0 (0.00 B)

## Model definition (Tensor Flow)

def build_qsc_MCD(X, l1_reg = 1e-6, dropout = 0.1):

X_in = Input(shape=[X.shape[1]])

X_phq = GatherLayer(phq_ind, axis=1, name='X_phq')(X_in)

X_gad = GatherLayer(gad_ind, axis=1, name='X_gad')(X_in)

X_sias = GatherLayer(sias_ind, axis=1, name='X_sias')(X_in)

X_pdss = GatherLayer(pdss_ind, axis=1, name='X_pdss')(X_in)

X_fq_ago = GatherLayer(fq_ago_ind, axis=1, name='X_fq_ago')(X_in)

X_fq_bld = GatherLayer(fq_bld_ind, axis=1, name='X_fq_bld')(X_in)

X_fq_soc = GatherLayer(fq_soc_ind, axis=1, name='X_fq_soc')(X_in)

X_fq_anx_dep = GatherLayer(fq_anx_dep_ind, axis=1)(X_in)

X_fq_global = GatherLayer(fq_global_ind, axis=1)(X_in)

X_fq_main = GatherLayer(fq_main_ind, axis=1)(X_in)

X_other = GatherLayer(other_ind, axis=1, name='X_other')(X_in)

D_phq = Dropout(dropout, name='D_phq')(X_phq)

L_phq = Dense(1, activation='linear', kernel_regularizer=L1(l1=l1_reg), kernel_constraint=NonNeg(), use_bias=False, name='L_phq')(D_phq)

D_gad = Dropout(dropout, name='D_gad')(X_gad)

L_gad = Dense(1, activation='linear', kernel_regularizer=L1(l1=l1_reg), kernel_constraint=NonNeg(), use_bias=False, name='L_gad')(D_gad)

D_sias = Dropout(dropout, name='D_sias')(X_sias)

L_sias = Dense(1, activation='linear', kernel_regularizer=L1(l1=l1_reg), kernel_constraint=NonNeg(), use_bias=False, name='L_sias')(D_sias)

D_pdss = Dropout(dropout, name='D_pdss')(X_pdss)

L_pdss = Dense(1, activation='linear', kernel_regularizer=L1(l1=l1_reg), kernel_constraint=NonNeg(), use_bias=False, name='L_pdss')(D_pdss)

D_fq_ago = Dropout(dropout, name='D_fq_ago')(X_fq_ago)

L_fq_ago = Dense(1, activation='linear', kernel_regularizer=L1(l1=l1_reg), kernel_constraint=NonNeg(), use_bias=False, name='L_fq_ago')(D_fq_ago)

D_fq_bld = Dropout(dropout, name='D_fq_bld')(X_fq_bld)

L_fq_bld = Dense(1, activation='linear', kernel_regularizer=L1(l1=l1_reg), kernel_constraint=NonNeg(), use_bias=False, name='L_fq_bld')(D_fq_bld)

D_fq_soc = Dropout(dropout, name='D_fq_soc')(X_fq_soc)

L_fq_soc = Dense(1, activation='linear', kernel_regularizer=L1(l1=l1_reg), kernel_constraint=NonNeg(), use_bias=False, name='L_fq_soc')(D_fq_soc)

D_fq_anx_dep = Dropout(dropout, name='D_fq_anx_dep')(X_fq_anx_dep)

L_fq_anx_dep = Dense(1, activation='linear', kernel_regularizer=L1(l1=l1_reg), kernel_constraint=NonNeg(), use_bias=False, name='L_fq_anx_dep')(D_fq_anx_dep)

L_fq_total_merge = Concatenate()([L_fq_ago, L_fq_bld, L_fq_soc])

D_fq_total = Dropout(dropout, name='D_fq_total')(L_fq_total_merge)

L_fq_total = Dense(1, activation='linear', kernel_regularizer=L1(l1=l1_reg), kernel_constraint=NonNeg(), use_bias=False, name='L_fq_total')(D_fq_total)

L_merge = Concatenate()([L_phq,L_gad,L_sias,L_pdss,L_fq_anx_dep,L_fq_total,X_fq_global,X_fq_main,X_other])

D_merge = Dropout(dropout)(L_merge)

output = Dense(4, activation='softmax', kernel_regularizer=L1(l1=l1_reg), name='Softmax')(D_merge)

model = Model(inputs=X_in, outputs=output)

model.compile(optimizer='adam', loss='categorical_crossentropy', metrics=['accuracy'])

return model
